# Young Adults with Negative Body Image at Fatness Subscale Are More Restrained Than Normal Adults during a Chocolate Discounting Task

**DOI:** 10.3390/ijerph20126122

**Published:** 2023-06-14

**Authors:** Sirui Huo, Jun Li, Jiaqi Guo, Han Yan, Xiaoyi Deng, Yong Liu, Jia Zhao

**Affiliations:** 1 Faculty of Psychology, Southwest University, Chongqing 400715, China; rui364591@email.swu.edu.cn (S.H.); cjv020304@email.swu.edu.cn (J.L.); swuguojiaqi@email.swu.edu.cn (J.G.);; 2 Key Laboratory of Cognition and Personality (Ministry of Education), Southwest University, Chongqing 400715, China; 3 Chongqing Collaborative Innovation Center for Brain Science, Chongqing 400715, China

**Keywords:** negative physical self, event-related potentials, chocolate discounting task

## Abstract

Research has confirmed that people with obesity exhibit special responses to food stimuli when it comes to food-related decision tasks. However, it is unclear whether the phenomenon exists in people who feel mentally obese, even though they are not obese. The aim of this study was to investigate the behavioral and neural correlations of food-related decision-making between young adults with negative body image at fatness subscale and a control group, so as to explore the differences in executive functioning between them. We used a time-delayed discounting task (DDT) and recruited 13 young female adults in each group to participate in the electroencephalogram (EEG) experiment. The number of selections for low immediate rewards and high delayed rewards was used as a performance indicator for DDT. Behavioral results showed a significant interaction effect between selection types and groups, where more delayed rewards and shorter immediate rewards were selected in the group with negative body image at fatness subscale than in the control group. Statistical correlations between body mass index (BMI) and selection times were found in the control group, but this phenomenon did not occur in the experimental group. The event-related potentials found that the P100 of young adults with a negative body image at fatness subscale was greater than those in the control group. P200 showed a significant interaction effect between groups, electrodes, and selection types. N200 and N450 in delayed rewards were more negative than in immediate rewards for both groups. These findings suggest that young adults with negative body image at fatness subscale are more restrained than young adults in the control group when choosing chocolates. Moreover, individuals with negative body image at fatness subscale might be more sensitive to food stimuli than individuals in the control group, because their P100 amplitude was significantly larger than that of individuals in the control group when exposed to food-related stimuli.

## 1. Introduction

In recent years, weight loss behavior among women has become prevalent under the guidance of “thinness is beauty”. However, in a recent study conducted in China, only 6.5% of female college students who were interested in losing weight actually had fat body types (i.e., those advised to lose weight), and the remainder mostly had blind weight loss (i.e., they did not have clear weight loss goal and did not use scientific weight loss methods) or excessive weight loss [1]. Another survey also showed that only 13.94% of female college students in China were actually overweight, and the prevalence of blind weight loss among college students was increasing year by year [2]. Additionally, physical and mental health problems related to weight loss are on the rise globally [3]. Disordered cognition may lead to abnormal eating behaviors such as anorexia nervosa and bulimia [4]. Numerous studies have shown that individuals often overestimate their size and weight, and therefore adopt the wrong eating strategies [4,5]. Having a clear and accurate perception of one’s weight is the key to adopting weight loss behaviors properly [6].

Fat-negative body image is defined as negative cognition, negative affective experiences, and corresponding behavioral modifications that result from an individual’s perception of obesity [7]. The term fat-negative body image can be used to describe the excessive weight loss behavior of female individuals even with normal weight (18 ≤ BMI < 23.5) who think they are fat (negative body image) due to anxiety about their bodies. People with a fat-negative body ego schema have an attentional bias toward body size and food stimulation, facilitating fat-negative information and showing selective preferences at different stages of information processing, such as attention, interpretation, and memory [8,9,10]. Studies of patients with eating disorders have suggested that the maintenance and development of this disorder are associated with an excessive focus on body appearance and negative attitudes toward people with obesity [11]. Some studies have shown that the use of social media exacerbates women’s negative perceptions of their own body size and weight [12]. However, there is no direct evidence about the cognitive mechanisms of adults with negative body image at fatness subscale.

Delayed gratification originated from a study by Walter Michel, who observed children’s processing of delayed food rewards in kindergarten [13]. It refers to the tendency of individuals to postpone immediate gratification and future outcomes in order to obtain more valuable long-term outcomes and higher rewards, which is influenced by individual self-regulation, individual decision-making styles, and self-control [14]. Delayed food gratification refers specifically to the times at which people postpone access to food to obtain more and higher-quality food. Food decisions are closely related to the functioning of the executive control network [15]. Past studies have mostly focused on physiological obesity, which is associated with impaired cognitive control in individuals [15,16]. Some delayed discounting task (DDT)-based studies have demonstrated that people with obesity allocate more cognitive resources [17], have poor self-control [18], and are more impulsive than healthy individuals when faced with food stimuli [13,14,18,19,20,21,22]. Considering the similar characteristics of negative body image at fatness subscale, the cognitive performance of people with obesity is of reference value for the study of normal-weight adults with negative body image at fatness subscale. In addition, based on our findings that young adults with obesity were more impulsive than normal-weight young adults during the food selection task [17], it should be reasonable to infer that normal-weight adults with a negative body image at fatness subscale are less likely to engage in impulse-induced overeating than individuals with physiological obesity.

Decision-making can be defined as the process of choosing between two or more competing alternatives; it requires a cost-and-benefit analysis of each option and an estimation of their consequences in the short, medium, and long term. The ability to control impulses is closely related to decision-making. In the threefold model of Patton et al., impulsiveness due to a lack of planning precisely reflects the tendency to make immediate decisions without evaluating the medium- and long-term consequences [23]. Thus, decision-making can be considered essential to an individual’s social adaptation, and is particularly difficult when there is a greater need to weigh immediate and future rewards and/or losses. From initial studies such as those by Bechara et al., the decision-making process has been studied by comparing the performances of patients with ventromedial prefrontal cortex lesions to those of healthy individuals, using the Iowa Gambling Task (IGT) [24]. The IGT is associated with various cognitive processes, including working memory, impulse control, probability estimation, and inverse learning [25].

In summary, there are no studies exploring the delayed gratification ability of people with negative body image at fatness subscale. Moreover, female individuals are more likely to engage in negative body image at fatness subscale than male individuals [26,27]; this is why we chose female adults in this pilot study. Thus, whether excessive attention to negative information causes negative body image, more self-control behaviors and more self-restraint in normal-weight female individuals with negative body image at fatness subscale is worth studying. For our initial study, female adults were chosen over males because females are more representative of negative body image at fatness subscale than male individuals. In order to investigate the correlation between the behavioral and neural performance of food-related decisions in adults with or without negative physical self-image, we combined DDT and event-related potential (ERP) studies in a population of female college students with normal BMI. Based on previous studies, we selected P100, P200, N200, and N450 as EEG features during the task, and made the following hypotheses: (1) individuals with negative body image at fatness subscale select less immediate/short-term chocolate than those individuals in the control group; (2) the individuals with negative physical self-perceptions are more sensitive to chocolate stimulus, and will think more about their choices, resulting in a high P1 amplitude for the early perception of stimuli, high P200 and N450 amplitudes for decision-making, and a low N200 amplitude for impulsivity during the later decision-making.

## 2. Materials and Methods

### 2.1. Participants

Some 26 female participants were recruited from Southwest University, Chongqing. All participants are 18–20 years old, and have normal or corrected-to-normal vision that is sufficient to complete the visual experiment. Taking into account their BMI and negative physical self scale (NPSS), they were divided into a group of 13 participants with negative body image at fatness subscale (average NPSS index > 2.5) and 13 control subjects (average NPSS index < 2), both with a normal BMI (18 ≤ BMI ≤ 23.5). None of them had any neurological or mental diseases, and they also did not take any kind of medication except water for four hours before the experiment. They also gave their written consent to participate in the experiment, and the study was approved by the Southwest University Ethics Committee.

### 2.2. Materials

#### 2.2.1. Self-Measurements

(1) Hunger and Desire to Eat

Participants rated their hunger and desire to eat by selecting a value from a 5-point visual-analog scale (VAS), rated from 1 to 5, indicating “not at all” to “very high”. This was an essential measure in this study because participants’ hunger states were strongly related to their performance during the food-related task [17,18]. The scale includes 8 questions, as follows: ① “What about your hunger degree now?”; ② “What about your thirst level now?”; ③ “How strong is your current desire to eat?”; ④ “How much food do you think you can eat right now? (In 100 g, and no more than 5 × 100 g)”; ⑤ “How full are you at the moment?”; ⑥ “How happy are you with your body right now?”; ⑦ “How happy are you at the moment?”; ⑧ “How sad are you at the moment?”. The mean value of the eight questions is used as the index of desire to eat for each participant.

(2) Dutch Eating Behavior Questionnaire—Restraint Scale (DEBQ-RS)

The Dutch Eating Behavior Questionnaire has 33 items for assessing people’s eating behavior, which can be divided into 3 subscales, including emotional eating, external eating, and restrained eating [17,28]. Here, the restraint scale of Dutch Eating Behavior Questionnaire with 10 items (e.g., “when your weight increases do you eat less than usual?”) is utilized to evaluate the participants’ diet behavior. Each item was rated on a 5-point scale from “never (1)” to “always (5)”, representing their restrained eating habits. In other words, participants with higher DEBQ-RS values showed greater control over eating. The subscale has been verified to have good internal consistency across weight category groups. In a nonclinical sample of participants with normal weight, overweight, and obesity, coefficient alphas ranged from 0.92 to 0.94 for the DEBQ-RS, from 0.96 to 0.97 for the DEBQ-emotional eating subscale, and from 0.79 to 0.84 for DEBQ-external eating subscale [29].

(3) Negative physical self scale at Fatness Subscale (NPSS-F)

The fat subscale of NPSS includes 11 items to measure affective, cognitive, and behavioral expression about body satisfaction; it was developed by Chen Hong et al. in 2006, and has a Cronbach’s alpha coefficient of 0.88 [18,27]. Five-point scales were set for each item (e.g., “I think I am fat in others’ eyes”), with an increasing trend from “never (0)” to “always (4)” representing their dissatisfaction with their body image. High NPSS-F scores indicate that participants are not satisfied with their body weight or consider themselves obese. In this study, people with scores of negative body image at fatness subscale averaged more than 2.0 in the NPSS-F questionnaire, which was consistent with the grouping criteria in previous studies [27,30].

(4) Power of Food Scale (PFS)

PFS was developed by Cappelleri et al. in 2009 to measure the psychological effects of food-abundant environments today, with a Cronbach’s alpha ranging from 0.81 to 0.91 [31]. It has 15 items and utilizes a 5-point scale for each item (e.g., “It seems like I have food on my mind a lot”), with an increasing trend from “totally disagree (1)” to “totally agree (5)” representing their appetite, i.e., their level of responsiveness to the food-abundant environment.

#### 2.2.2. Chocolate-Related Delayed Discounting Task

**Figure 1:**
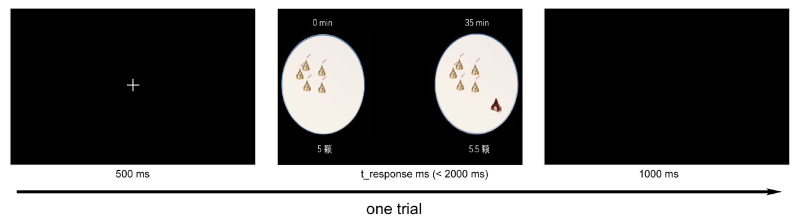
One trial from the chocolate-related delayed discounting task, where 5 and 5.5 chocolates displayed in left and right of stimulus material respectively. “颗” was the quantifier used to measure the amount of chocolate.

To study the neural and behavioral responses of young adults with negative body image at fatness subscale, a chocolate-related DDT was employed [17,32]. Using “as if” situations in the experiment, participants were asked to choose one chocolate reward from two selections with different time intervals and amounts of chocolate (i.e., smaller immediate rewards or larger delayed rewards), where they would not obtain real chocolate after the experiment [33,34]. The experimental procedure for one trial is shown in Figure 1. A fixation was displayed first (500 ms), then a stimulus picture with two kinds of chocolate rewards (≤2000 ms) appeared until the participants pressed the selection key. After the stimulus picture, a black screen was presented for 1000 ms to allow enough time intervals between trials. The smaller immediate rewards were set with 5 chocolates and a 0 min delay, while the larger delayed rewards were set with different combinations (the amount of chocolate varied from 5.5 to 20; the delay time varied between 14, 26, 35, 50, 65, and 90 min), similar to the parameters of previous studies [35]. The participants were required to press “F” when choosing short and immediate rewards, and press “J” to choose large and delayed rewards; the keys were counterbalanced between two sessions for each participant. Each session has 180 trials with 2 min of rest between the two sessions.

#### 2.2.3. Procedure

To reduce the disturbance of satiety and hunger, participants were asked not to eat anything in the 3 h before the start of the experiment [36,37]. Their body weight and height were measured to calculate BMI after they signed their informed consent. Then, several questionnaires, including hunger score, appetite, PFS, and DEBQ-RS, were collected from them. After that, the chocolate-related DDT was performed, with their EEG recorded simultaneously. The female participants were recruited through an electronic advertisement from Southwest University several days before the experiment, and there were no sequences arranged between the two groups, i.e., a pseudorandom order for the participants.

#### 2.2.4. Data Recording and Analysis

(1) Behavior Analyses

The mean value (M) and standard deviation (SD) were calculated for the self-reported information from two groups, including their age, DEBQ-RS, NPSS-F, PFS, hunger, desire to eat, and BMI. Meanwhile, the differences in these scores between the two groups were compared with independent sample *t*-tests in SPSS software 19 (IBM, Armonk, NY, USA). Further, the selection times for small immediate rewards and large delayed rewards were compared between groups using a repeated-measure analysis of variance (ANOVA). Simple effect tests were also applied to selection times, as a significant interaction effect was found between groups and selections. Finally, a Pearson correlation analysis was applied to BMI and selection times. All analyses were conducted using SPSS 19.

(2) EEG Recording and Analyses

EEG data were recorded with 32 Ag/AgCl scalp electrodes using the NeuroScan 7181 series (Compumedics, Clarlotte, NC, USA). The sampling rate of EEG equipment was 1000 Hz. EEG data were analyzed using EEGLAB v2022.0 (Swartz Center for Computational Neuroscience, La Jolla, CA, USA) [38], an open source toolbox running on MATLAB software R2022a (The MathWorks, Inc., Natick, MA, USA). Continuous EEG data were filtered by a finite impulse response (FIR) filter with a bandpass between 1 and 30 Hz. Following that, EEG trials were extracted (−500 ms to 1000 ms) and visually inspected from trial to trial. Trials of EEG data with much larger amplitudes or chaotic fluctuations were excluded from the initial trials. In the meantime, trials with response times outside of (250, 1950 ms) seemed like half-hearted selections and were discarded. After that, an independent component analysis (ICA) was applied to the remaining trials to remove eye blinks, head movements, and channel noise from the data for each participant [38]. After removing the independent components, the trials were inspected again to ensure that there were no bad trials remaining [39]. Then, the grand-averaged ERP activities were calculated for groups (negative body image at fatness subscale/control) and selections (immediate reward/delayed reward), with a reference baseline between −100 ms and 0 ms, respectively. Based on previous studies and our experimental design, several ERP components including P100 (30–50 ms), N200 (255–290 ms), N450 (420–510 ms), and late positive component (LPC) (580–630 ms) were compared between different groups and selection types.

After the above analyses, we examined the participants’ personal information and behavioral performance, and removed three participants’ EEG data (one’s BMI was lower than 18, one’s selections were all delayed reward, and one participant took part in the experiment twice in a short time interval as she was uncomfortable and was not finished with the experiment the first time). Therefore, 13 undergraduates with negative body image at fatness subscale and 13 normal-control undergraduates were retained for the following analyses.

Finally, SPSS 19 was utilized for the statistics process, where a three-way repeated-measures ANOVA was applied to the mean amplitudes of P100, P200, N200, N450, and LPC, respectively, resulting in a 2 (groups of subjects: negative body image at fatness subscale/normal control) × 2 (selection types: immediate reward/delayed reward) × 5 (scalp position of electrodes: Fz/FCz/Cz/Cpz/Pz) ANOVA for each component. Finally, either the post-doc analysis or a simple effects analysis was also tested with SPSS, according to the ANOVA results.

## 3. Results

### 3.1. Behavioral Performance

(1) Self-Reported Results

Table 1 displays the grand-average results for ERPs and scalp topography under different conditions. The statistical results for the four ERP components are shown below. DEBQ-RS (*t* = 6.21, *p* < 0.001). NPSS (*t* = 11.52, *p* < 0.001).

**Figure 2:**
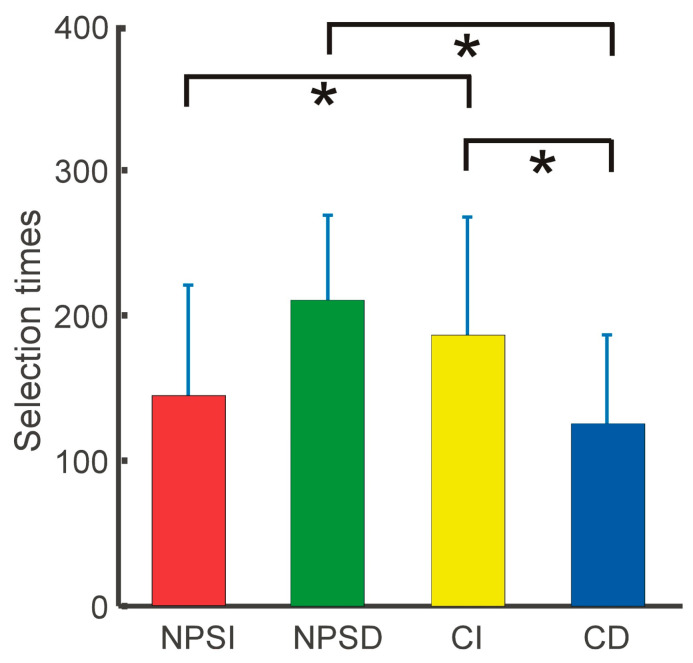
Simple effect tests for the two selection types (immediate/delayed) of two groups (group with negative body image at fatness subscale/control group). NPSI: NPSS group with immediate selections; NPSD: NPSS group with delayed selections; CI: control group with immediate selections; CD: control group with delayed selections.* indicates *p* < 0.05.

Statistical analysis showed that the interaction between selection types and groups was significant (F = 5.48, *p* = 0.028). In simple effect tests (Figure 2), the immediate selection times for the group with negative body image at fatness subscale were significantly shorter than those for the control group (*p* = 0.020), but the delayed selection times were significantly larger (*p* = 0.041). In addition, the immediate selections were significantly more than the delayed selections for the control group (*p* = 0.039), but this effect did not appear in the group with negative body image at fatness subscale.

(2) Correlation between BMI and Selection Times

**Figure 3:**
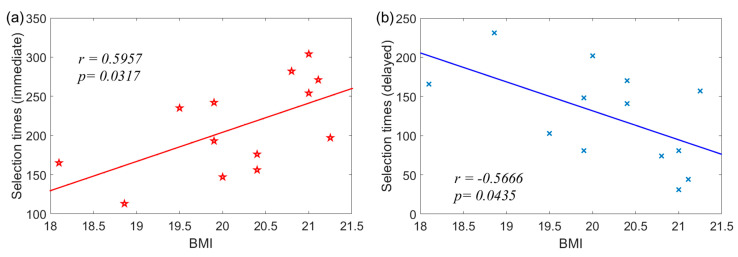
Correlation between behavioral performance and BMI of control group: (**a**) immediate selections; (**b**) delayed selections.

Figure 3 displays a positive correlation between the immediate selection times and BMI of the control group. In contrast, there is a negative correlation between delayed selection times and BMI; however, these correlations are not found in the group with negative body image at fatness subscale.

### 3.2. ERP Results

**Figure 4:**
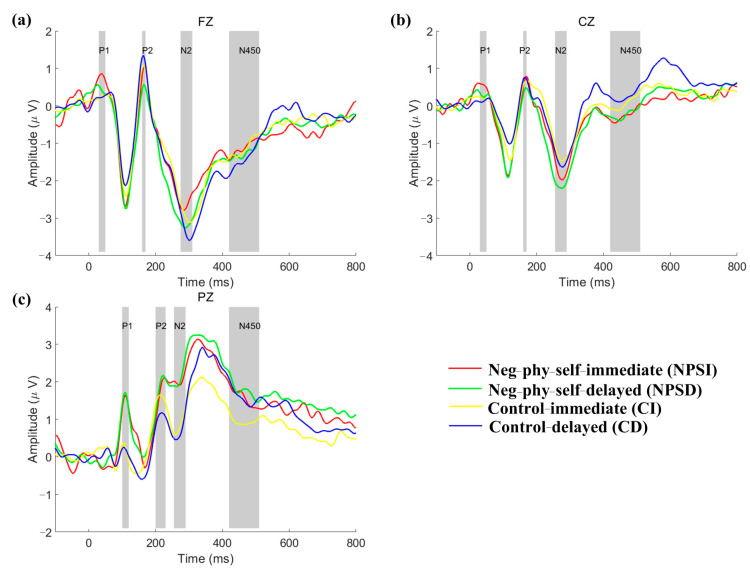
Event-related potentials for the negative body image group and control group. (**a**–**c**) correspond to ERPs at Fz, Cz, and Pz electrodes, respectively. Gray bar indicate the selected time range for each ERP component.

**Figure 5:**
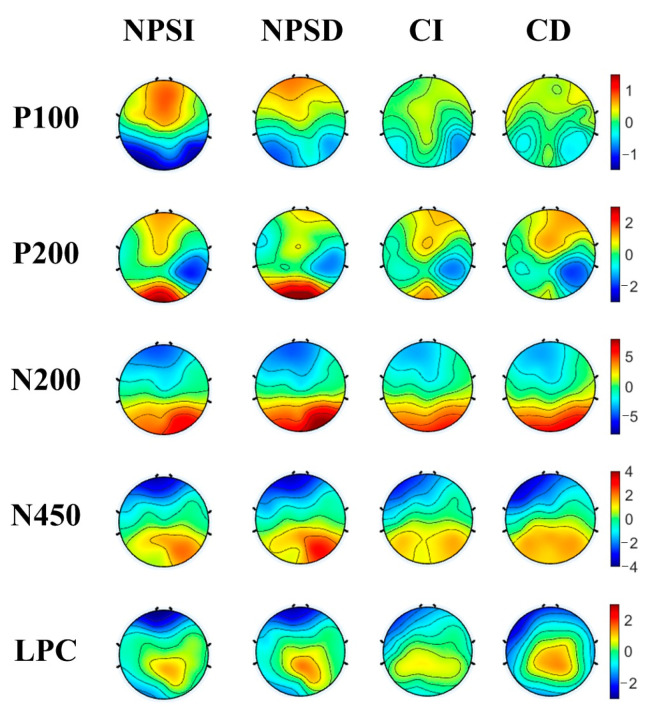
Topography plots for negative body image group and control group of five ERP components (P100, P200, N200, and N450).

Figure 4 and Figure 5 display the grand-average results for ERPs and scalp topography under different conditions. The statistical results for the four ERP components are shown below.

**P100** **:**

A repeated-measures ANOVA on P100 showed a significant main effect between groups (F = 4.362, *p* = 0.048), where no other significant effects were found. 

Post-doc tests found that the amplitude of P100 for the group with negative body image at fatness subscale was significantly larger than that of the control group.

**P200:**

A repeated-measured ANOVA on P200 showed significant interaction effects between groups, selection types, and electrodes (F = 3.155, *p* = 0.037), where no other significant effects were found for P200.

The simple effects analysis indicated that the amplitude of P200 at Fz was greater (marginally significant) than the delayed selection (*p* = 0.058) in the group with negative body image at fatness subscale, but not in the control group. In addition, the P200 under immediate selection was significantly larger (marginally) than that under delayed selection for the control group (*p* = 0.054) at Cpz.

**N** **200:**

A repeated-measured ANOVA on N200 showed a significant main effect between selection types (F = 5.418, *p* = 0.029) and a significant main effect between electrodes (F = 34.684, *p* < 0.001), where no other significant effects were found.

Post-doc tests on the selection types showed that N200 under the delayed selection type was more negative than that under the immediate selection type. Post-doc tests showed that N200 between any two electrodes was significant different, and their amplitudes were in the order of Fz < FCz < Cz < Cpz < Pz, i.e., N200 at Fz was the most negative in five electrodes.

**N450:**

A repeated-measured ANOVA on N450 showed a significant main effect between selection types (F = 21.818, *p* < 0.001), a significant main effect between electrodes (F = 32.356, *p* < 0.001), and a significant interaction effect between electrodes and selection types (F = 13.089, *p* < 0.001), where no other significant effects were found. 

A simple effects analysis showed that the mean amplitude of N450 under the delayed rewards was significantly negative at Fz (*p* < 0.001), Fcz (*p* < 0.001), Cz (*p* < 0.001), and marginally significant at Cpz (*p* = 0.075), more so than that under the immediate rewards. Post-doc tests showed that N450 under the delayed selections was significantly more negative than that under the immediate selections. Post-doc tests for N450 at different electrode positions revealed that there were significant differences between the mean amplitudes of any two electrodes, and they were in the order of Fz < FCz < Cz < Cpz < Pz.

## 4. Discussion

As predicted, young female adults with negative body image at fatness subscale showed behavioral and ERP variations compared to adults in the control group. It was found that the experimental group was more restrained with food than the control group, as they selected fewer immediate rewards during the DDT task. Similarly, the ERPs of adults with negative body image showed different variations from those of adults in the control group.

An interaction effect was found among groups and selection times. Young adults with negative body image selected more delayed rewards than immediate rewards, while the control group selected more immediate rewards than delayed rewards. The selection results here were consistent with our previous hypothesis. Compared to the control group, adults with negative body image typically limit their food intake to improve their body satisfaction, just as restrained eaters do. NPSS-F and DEBQ-RS ratings of young adults with negative body image showed restrained eating behavior as well (Table 1). Interestingly, the selection times for immediate rewards/delayed rewards were positively/negatively correlated with BMI in control individuals (Figure 3), but these correlations were not found in the experimental group. The correlation results here were meaningful because they suggested that young adults with high BMI were more likely to select immediate rewards, but females with lower BMI showed the reverse performance. This was similar to our previous results, which showed that adults with obesity had a stronger impulse to choose more immediate rewards during the DDT task [17]. In the experimental group, the absence of a correlation with impulsivity might be attributed to their negative body image, which may prevent them from selecting immediate rewards as much as they want. Other self-reported information, such as age, PFS, hunger, and desire to eat, should have a minimal effect on influencing their selection times for the two conditions, as no correlation was found between them.

P100 was related to physical perception attention [40,41]. In this study, it was found that the two groups allocated different attention resources to the chocolate stimuli in the DDT task, as the P100 amplitude of adults with negative body image was much larger than that of control adults. In contrast, the control subjects did not show a noticeable P100 in Fz and Cz, and only Pz displayed a significant P100 fluctuation in visual stimuli. Females with negative body image might be more vigilant to chocolate stimuli (high-energy foods) than control adults, as the P100 at Fz, Cz, and Pz electrodes was more pronounced than control adults. The reasons for the distinct P100 could be explained below. Adults with fat-negative physical self-perceptions had a negative body image and restrained eating behavior, which was related to high-energy food inhibition. Additionally, the initial allocation of attention would seem to affect the response time and reward selection neural process for both groups.

Previous studies have linked P200 to selective attention in decision-making [41,42], and N200 to impulsivity [17]. One study found that the P200 of people with obesity was larger in food-Stroop tasks than that in the control group, indicating more attention allocation to food stimuli in people with obesity [43]. The greater P200 of people with obesity to food-related stimuli was reconfirmed in our previous study of a food go/no-go task [18]. Meanwhile, an ERP study with DDT showed that delayed rewards evoked greater N200 than immediate rewards [44]. Another study showed that the N200 of people with obesity for delayed rewards was greater than for immediate rewards [17]. In this study, the P200 for immediate rewards was larger than for delayed rewards at Fz in the group with negative body image. Given the shared characteristics (high NPSS index) between adults with obesity and normal-weight adults with highly negative body image, it was easily understood that the experimental group might pay more attention to immediate rewards due to their mental burden of negative body image and restrained eating behavior. Likewise, the N200 under delayed rewards was much greater than those under immediate rewards for both groups, but no group differences were found. Thus, individuals with negative body image should have similar attentional biases to food stimuli as people with obesity, but they were more restrained than people with normal weight or obesity during the selection of two types, resulting in no significant N200 between groups.

N450 represents a response conflict to stimuli, where larger conflicts evoke greater amplitude [45,46,47]. In this study, the amplitude of N450 was the most negative in the frontal cortex, followed by the medial cortex, and finally the occipital cortex, which was consistent with the previous statements that frontal-central locations were most pronounced [47]. Meanwhile, the DDT task also showed a higher amplitude under delayed rewards, suggesting that the participants needed more cognitive resources to counterbalance the delayed time and amounts of rewards.

Furthermore, both ERPs and behavior here differ from our previous DDT study with young adults with obesity [17], which may be related to the differences in psychological and physiological factors between the two studies. The psychology of “high dietary restraint” or “excessive weight concern” was the main concern of individuals with negative body image. Individuals with negative body image may be overly concerned about their weight due to a negative body image, and show a stronger desire to restrain themselves from eating (without binge eating) than adults with obesity. Although the experimental group here showed a consistent tendency toward negative physical self perception compared with individuals with physiological obesity, they were more restrained in food intake, as their DEBQ-RS score was higher than that of adults with obesity in our previous study [17]. Individuals with negative body image may be overly concerned about their weight due to a negative body ego. This may manifest as a strong desire to restrain eating, and the desire to restrain is stronger than that in overweight adults. Moreover, the difference between individuals with negative body image and individuals with physiological obesity was also displayed in the flexibility or strictness of self-imposed restraint, where strict restraint was associated with higher BMI and weight loss failure, while highly flexible restraint was associated with a lower BMI and weight loss success [48]. Both individuals with physiological obesity and individuals with negative body image have a negative body ego and the same level of the need to lose or maintain weight. However, individuals with physiological obesity may correspond to strict restraint, and individuals with negative body image may correspond to flexible restraint. Another piece of evidence supporting this hypothesis is that strict restraint is associated with slower responses to food-related stimuli and with increased cognitive load for food-related stimuli, whereas no such effect exists for flexible restraint [49]. This may, to some extent, explain the differential performance of individuals with negative body image versus individuals with physiological obesity on behavioral tests.

Finally, there are still some limitations that need to be explored in the future. Firstly, the sample size was relatively small. Due to the influence of COVID-19 last year, it was difficult to complete the experiment with more participants, which may have influenced the reliability and expandability of the results here. Secondly, the participants were recruited based on their BMI and NPSS-F index, which omitted other alternative anthropometric measurements, such as abdominal circumference, waist–hip ratio, and so on. The lack of these measurements limits the possibility of exploring more relationships between participants’ phenotypes and electrophysiological fluctuations. Thirdly, the duration of possible medication use was too short, and the level of alcohol abuse and drug consumption by the participants was not recorded during the experiment. We neglected this point because the participants were female undergraduates at Southwest University, where students rarely abuse alcohol or consume psychoactive substances. However, this omission reduces the reliability of the results here, as these variables play critical roles in executive performance and decision-making. Finally, the experiment used chocolate as the stimulus material, which may not be representative. With more kinds of food as stimulus materials, the experimental results might be more general and meaningful for detecting executive functioning in young adults with high NPSS-F.

## 5. Conclusions

In summary, young female adults with negative body image at fatness subscale exhibited strongly restrained behavior with regard to chocolate, with fewer choices of immediate rewards; this result was different from that of female adults in the control group. ERP results showed that individuals with negative body image were more sensitive (with a larger P100 amplitude in the early period after stimuli) to chocolate stimuli than individuals in the control group. Additionally, the late components (P200, N200, and N450) also displayed different time-spatial fluctuations between immediate rewards and delayed rewards.

## Figures and Tables

**Table 1:** Demographic information and self-reported results.

Variable	Group with Negative Body Image at Fatness Subscale (M ± SD) *N* = 13	Control Group (M ± SD) *N* = 13
Age	19.21 (1.31)	18.85 (1.07)
DEBQ-RS ***	38.07 (7.35)	22.62 (5.33)
NPSS-F ***	2.92 (0.30)	1.62 (0.28)
PFS	55.00 (11.57)	48.69 (6.22)
Hunger	3.43 (1.95)	4.08 (1.27)
Desire to eat	2.03 (0.37)	2.13 (0.47)
BMI	20.81 (1.62)	20.64 (2.03)

Note: *** *p* < 0.001.

## Data Availability

The data presented in this study are available on request from the corresponding author. The data are not publicly available due to concerns about privacy and ethics in personal decision-making.
